# Monocytes at the crossroads of inflammation and labor: biomarker potential in prolonged deliveries – a narrative review

**DOI:** 10.1097/MS9.0000000000004063

**Published:** 2025-10-09

**Authors:** Emmanuel Ifeanyi Obeagu

**Affiliations:** Department of Biomedical Laboratory Science, Africa University, Mutare, Zimbabwe

**Keywords:** biomarkers, cytokines, immune response, monocytes, prolonged labor

## Abstract

Prolonged labor is a significant obstetric complication, often leading to adverse maternal and neonatal outcomes. Recent research has focused on the role of the immune system, particularly monocytes, in the pathophysiology of prolonged labor. Monocytes contribute to the inflammatory processes that govern cervical ripening, uterine contractility, and labor progression. However, dysregulation in monocyte activation and cytokine release can impair these processes, leading to labor complications. This review examines the role of monocytes as biomarkers in prolonged labor, emphasizing their involvement in immune response modulation and the potential for predicting labor outcomes. Monocytes are central to the inflammatory response during labor, where they are recruited to the cervix and uterus and release pro-inflammatory cytokines that facilitate cervical softening and uterine contractions. Changes in monocyte subsets and the cytokines they produce have been linked to both normal and pathological labor. Dysregulation in this immune response, characterized by either an overactive or underactive monocyte function, can result in prolonged labor. Identifying monocyte-derived biomarkers such as specific cytokine profiles and monocyte surface markers could offer valuable insights into the timing and progression of labor, helping to predict complications before they occur.

## Introduction

Prolonged labor is a significant obstetric complication that increases the risk of maternal and neonatal morbidity and mortality. It is defined as labor that extends beyond the expected duration, typically lasting more than 18–24 hours in nulliparous women and over 12 hours in multiparous women. This extended labor process can lead to increased risk of fetal distress, infection, uterine rupture, and other complications that require timely intervention. The causes of prolonged labor are multifactorial, ranging from mechanical and biological factors to maternal health conditions and inadequate uterine contractions. One crucial yet underexplored factor that may contribute to its pathogenesis is the immune response, particularly the activation of monocytes and their associated cytokine production^[1–4]^. Monocytes are a type of white blood cell that play a vital role in the innate immune system. They serve as precursors to macrophages and dendritic cells, both of which are central to the body’s inflammatory response. During labor, monocytes migrate to the uterus and the cervix, where they contribute to the inflammatory cascade that facilitates cervical ripening and uterine contractions. However, when this immune response is dysregulated, it can lead to excessive inflammation, contributing to complications like prolonged labor. Understanding how monocytes behave during labor and how they may contribute to its protracted course is crucial for identifying potential biomarkers and therapeutic targets^[5–7]^. Emerging research suggests that monocyte subsets exhibit distinct functional roles during labor. These subsets, including classical, intermediate, and nonclassical monocytes, vary in their inflammatory potential and ability to migrate to tissues involved in labor progression. In prolonged labor, the recruitment and activation of these monocytes may be altered, leading to abnormal cytokine profiles that hinder normal labor progression. For instance, an overactive inflammatory response driven by monocytes could impede uterine contractions, delay cervical dilation, and increase the risk of infection^[8–10]^.

**HIGHLIGHTS**

- *Role in Inflammation*: Monocytes regulate inflammation, offering insights into prolonged labor pathophysiology.
- *Biomarker Potential*: Elevated monocyte activity may indicate labor complications.
- *Immune Modulation*: Monocytes mediate maternal-fetal immune responses.
- *Therapeutic Targets*: Emerging therapies focus on monocyte-driven pathways.
- *Research Gaps*: Future studies should explore monocyte heterogeneity and predictive value

The cytokine profiles produced by monocytes during labor are also crucial for regulating the onset and progression of labor. Cytokines like interleukin-1β (IL-1β), tumor necrosis factor-alpha (TNF-α), and interleukin-6 (IL-6) play key roles in promoting uterine contractions and cervical remodeling. Dysregulation of these cytokines, often seen in prolonged labor, can lead to an imbalance between pro-inflammatory and antiinflammatory cytokines, creating an environment that is not conducive to normal labor progression^[11–13]^. Given these insights, monocyte-based biomarkers present an intriguing potential for diagnosing and managing prolonged labor. By measuring the levels of specific cytokines or identifying unique monocyte subsets in maternal blood, clinicians may be able to predict the risk of prolonged labor early on, enabling timely interventions. However, the clinical translation of these biomarkers remains a challenge, as the detection methods must be both sensitive and specific, and must account for preanalytical factors like sample collection and processing^[14–16]^. Furthermore, monocyte biomarkers could serve as predictive tools for assessing the risk of complications during labor. For example, identifying elevated levels of certain cytokines or the presence of activated monocytes could signal impending complications, such as fetal distress or maternal infection, allowing for early intervention and potentially reducing the need for more invasive procedures like caesarean sections. This shift toward a more personalized approach in labor management could significantly improve maternal and neonatal outcomes[17]. This review aims to critically evaluate the role of monocytes as biomarkers in prolonged labor, discussing their functions, cytokine profiles, and the challenges in translating these findings into clinical practice.

## Aim

The aim of this review is to provide a comprehensive overview of the role of monocytes as biomarkers in prolonged labor.

## Rationale of the review

Prolonged labor is a common obstetric complication associated with significant maternal and neonatal risks, including increased rates of cesarean delivery, maternal morbidity, and neonatal distress. Despite advances in obstetric care, the mechanisms underlying prolonged labor remain poorly understood, making early identification and effective management challenging. The inflammatory response, particularly the involvement of immune cells such as monocytes, plays a crucial role in the processes of labor, including cervical ripening, uterine contractility, and the onset of labor^[18,19]^. Monocytes are pivotal in modulating inflammation and immune responses during labor, and dysregulation of their function has been implicated in the pathophysiology of prolonged labor. Research suggests that alterations in monocyte subsets, cytokine production, and activation patterns may contribute to labor dysfunction, making them a promising biomarker for predicting and managing prolonged labor. However, the current understanding of their role in labor remains fragmented, with limited clinical application of monocyte-based biomarkers for diagnosing or predicting labor progression^[20,21]^. This review is motivated by the need to synthesize current insights into the involvement of monocytes in prolonged labor and explore their potential as biomarkers for improving clinical outcomes. By identifying monocyte-driven cytokine imbalances, activation patterns, and immune dysregulation, the review aims to provide a more thorough understanding of the underlying mechanisms of prolonged labor. Additionally, it seeks to highlight the challenges and future directions in translating monocyte-based biomarkers into routine clinical practice, ultimately paving the way for better-targeted interventions and personalized approaches to managing prolonged labor^[22,23]^.

## Review methods

The methods employed in this narrative review were designed to comprehensively examine the current understanding of monocytes as biomarkers in prolonged labor. To ensure a thorough exploration of this emerging topic, we adopted a systematic approach in selecting and synthesizing relevant studies from both clinical and experimental research. The review followed established guidelines for narrative reviews, with a focus on monocyte subsets, cytokine profiles, and their potential as predictive tools for prolonged labor. The narrative review started by conducting an extensive literature search across multiple databases, including PubMed, Scopus, and Web of Science, using a range of search terms such as “monocytes,” “prolonged labor,” “inflammation,” “cytokines,” “immune response,” and “biomarkers in labor.” Studies were selected based on their relevance to monocyte function during labor, their exploration of immune responses in the context of prolonged labor, and their investigation of diagnostic or therapeutic implications. The inclusion criteria for the review focused on original research articles, including both human and animal studies that discussed the immune mechanisms underpinning prolonged labor. In particular, studies were considered that provided insights into the role of monocyte activation, cytokine production, immune signaling pathways, and their connection to the protracted progression of labor. We also included studies that evaluated the use of monocyte-based biomarkers for predicting labor outcomes or guiding clinical interventions. Exclusion criteria included articles that primarily focused on nonimmune factors in prolonged labor, such as maternal age, birth canal abnormalities, and mechanical causes of labor delay, as these fell outside the scope of this review. Furthermore, we excluded articles that were not published in English or those lacking substantial data on monocyte biomarkers.

Following the initial search, selected studies were carefully reviewed to assess the quality and reliability of their findings. A qualitative analysis was performed to identify key themes related to monocyte function in prolonged labor, including the mechanisms of monocyte recruitment, activation, and cytokine production during labor. We also focused on studies that compared monocyte-based biomarkers with other conventional diagnostic markers for prolonged labor, such as cervical ripening scores or uterine contraction monitoring. The synthesis of findings was guided by the objective of identifying the gaps in knowledge and areas where future research could enhance our understanding of the role of monocytes in labor progression. In particular, we sought to explore the limitations of current biomarkers and diagnostic approaches and to identify the practical challenges involved in translating monocyte profiling into clinical practice. Throughout the review process, we adhered to ethical considerations, ensuring that studies involving human subjects were conducted in accordance with ethical guidelines and informed consent procedures. The findings of the review were structured in a manner that highlights both the potential applications and limitations of monocyte-based biomarkers in clinical settings, providing a balanced and evidence-based overview of the field.

## Monocyte recruitment and activation during labor

**Figure 1.**
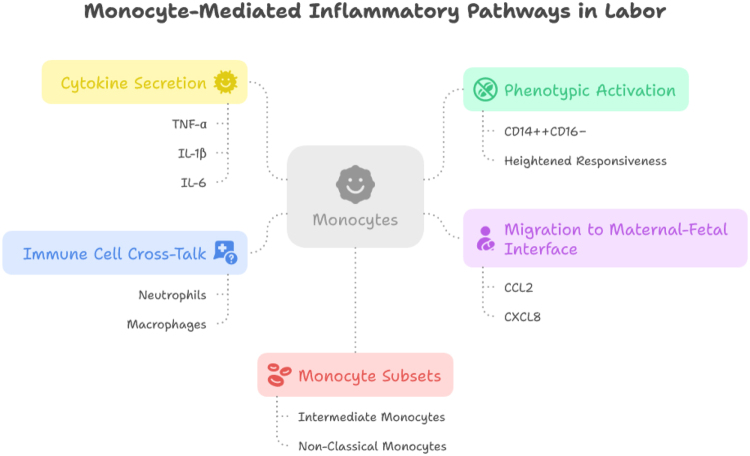
Monocyte-mediated inflammatory pathways during normal labor.

Monocyte recruitment and activation play a crucial role in the initiation and progression of labor. Monocytes, which are circulating white blood cells, are part of the body’s innate immune response and serve as key mediators in inflammatory processes. During labor, they are recruited to the cervix and uterus, where they contribute to cervical ripening, uterine contractility, and the overall inflammatory milieu necessary for childbirth. Their activation, both in terms of cellular recruitment and functional changes, is integral to the physiological process of labor. This process is driven by several molecular signals, including hormonal and cytokine mediators, that alter the behavior of monocytes during labor^[24,25]^. At the onset of labor, a series of signaling events trigger the recruitment of monocytes to the cervix and uterus. These signals include hormonal changes such as the release of prostaglandins (PGs), as well as the production of cytokines and chemokines from local tissues. These mediators act as chemotactic factors, guiding monocytes from the bloodstream into the uterine and cervical tissues. For instance, cytokines such as IL-1β, TNF-α, and IL-6 are produced in response to uterine contractions and cervical remodeling. These cytokines activate endothelial cells within blood vessels, allowing monocytes to adhere to the vessel walls and transmigrate into the surrounding tissues. This recruitment is essential for the inflammatory processes that facilitate cervical ripening and the establishment of uterine contractions^[26,27]^. Once monocytes enter the cervix and uterus, they undergo further activation, differentiating into macrophages and other immune cells that participate in labor processes. Upon activation, monocytes secrete a range of pro-inflammatory cytokines, including IL-1β, TNF-α, IL-6, and interleukin-8 (IL-8), which promote tissue remodeling, cervical softening, and uterine contractions. These cytokines also contribute to the breakdown of extracellular matrix (ECM) components, allowing for cervical dilation and effacement. Additionally, monocytes produce matrix metalloproteinases (MMPs), enzymes that play a pivotal role in tissue degradation, which is required for cervical dilation. Through these actions, monocytes help orchestrate the physiological events that allow for the progression of labor[28]. The recruitment and activation of monocytes are tightly regulated to ensure that labor progresses in a controlled manner. An overactive or dysregulated inflammatory response can lead to complications such as preterm labor, uterine hyperstimulation, or prolonged labor. In cases where monocyte activation is insufficient or delayed, labor may be prolonged due to inadequate cervical ripening and weak uterine contractions. Therefore, a delicate balance of monocyte recruitment and activation is essential for the timely progression of labor. Monitoring the dynamics of monocyte recruitment and activation could potentially serve as a diagnostic tool for assessing labor progression and predicting complications (Fig. 1)^[29,30]^.

## Monocytes as biomarkers for prolonged labor

Monocytes, due to their pivotal role in immune responses, have garnered increasing attention as potential biomarkers for monitoring and predicting prolonged labor. Prolonged labor is defined as labor that exceeds the expected time frame for progression, and it is associated with various complications, including increased risk of cesarean section, maternal infections, postpartum hemorrhage, and fetal distress. Traditional clinical markers, such as cervical dilation and uterine contraction patterns, provide limited predictive power for complications, particularly in cases of prolonged labor. As a result, there has been growing interest in identifying biomarkers that can offer more precise insights into the mechanisms driving labor progression and the onset of complications[31]. Monocytes play a central role in the inflammatory response during labor. At the onset of labor, monocytes are recruited to the cervix and uterus, where they are activated and differentiate into macrophages. These immune cells secrete pro-inflammatory cytokines that are essential for cervical ripening, uterine contractions, and the initiation of labor. Dysregulation of this immune response, however, can impair cervical softening and uterine contractility, leading to prolonged labor. The changes in the number, activation status, and cytokine profiles of monocytes during labor may serve as valuable indicators of labor progression, offering new opportunities for early diagnosis and intervention^[32,33]^. The role of monocytes as biomarkers for prolonged labor is primarily based on their ability to produce and release a variety of cytokines that influence labor dynamics. Studies have shown that women experiencing prolonged labor exhibit altered monocyte subsets and cytokine profiles compared to those with normal labor. For instance, increased levels of pro-inflammatory cytokines such as IL-1β, TNF-α, and IL-6 have been associated with prolonged labor, indicating a heightened inflammatory response. Additionally, an increased proportion of activated monocytes, as measured by surface markers such as CD14 and HLA-DR, has been observed in women with prolonged labor. These alterations in monocyte function suggest that monocyte-driven inflammation may contribute to the failure of normal labor progression^[34,35]^.

Monocytes’ ability to act as biomarkers for prolonged labor is further supported by their dynamic changes in response to labor-related signals. During normal labor, monocytes exhibit a controlled activation and cytokine release, facilitating the necessary tissue remodeling and uterine contractions. In cases of prolonged labor, however, this process becomes dysregulated, resulting in either excessive or insufficient inflammatory responses. Monitoring specific monocyte markers, such as the expression of activation markers (e.g., CD16 and CD14) and cytokine production profiles, could provide a noninvasive method for assessing the risk of prolonged labor. These biomarkers could potentially be used to predict labor duration and complications, allowing clinicians to make more informed decisions regarding labor management and the timing of interventions[36]. Despite the promising potential of monocytes as biomarkers for prolonged labor, several challenges remain in translating these findings into clinical practice. First, there is a need for standardization in the methods used to measure monocyte subsets and cytokine profiles in maternal blood or cervicovaginal secretions. Current studies have employed a variety of techniques, such as flow cytometry and cytokine assays, but further research is required to determine the most reliable and reproducible markers for labor progression. Additionally, the clinical utility of monocyte-based biomarkers needs to be validated in larger, diverse populations to ensure their predictive accuracy across different patient groups[37].

## Cytokine imbalances and monocyte dysregulation in prolonged labor

Cytokine imbalances and monocyte dysregulation are central contributors to the pathophysiology of prolonged labor. Labor is a complex process that requires precise coordination between the immune system, hormonal regulation, and tissue remodeling. Cytokines, small signaling proteins produced by immune cells like monocytes, orchestrate these processes by modulating inflammation, smooth muscle contraction, and tissue breakdown. In normal labor, a delicate balance of pro-inflammatory and antiinflammatory cytokines is necessary for cervical ripening, uterine contractility, and successful delivery. However, when this balance is disrupted, particularly by monocyte dysfunction, labor progression can be impaired, leading to prolonged labor and associated complications[38]. Monocytes are a major source of pro-inflammatory cytokines, such as IL-1β, TNF-α, IL-6, and IL-8, which are known to be involved in cervical remodeling and uterine contractility. During normal labor, monocytes are recruited to the cervix and uterus, where they differentiate into macrophages and secrete cytokines that facilitate the breakdown of ECM components, softening the cervix and preparing the uterus for contractions. These cytokines also play a crucial role in the initiation of uterine contractions by inducing the synthesis of PGs, which stimulate smooth muscle activity. However, when cytokine production becomes dysregulated, either by overproduction or insufficient release, the normal labor process can be disrupted, resulting in prolonged labor^[39,40]^. In cases of prolonged labor, an imbalance in cytokine levels is often observed. Specifically, elevated levels of pro-inflammatory cytokines, such as IL-1β, TNF-α, and IL-6, have been linked to an exaggerated inflammatory response that leads to uterine hyperstimulation and cervical dystocia. On the other hand, insufficient activation of these cytokines, or a dominance of antiinflammatory cytokines such as interleukin-10 (IL-10), may result in inadequate cervical ripening and weak uterine contractions. The failure of these cytokines to properly coordinate the inflammatory response and tissue remodeling may significantly delay labor progression. Furthermore, monocyte activation, as indicated by surface markers like CD14 and HLA-DR, is often altered in women with prolonged labor, suggesting a dysfunction in monocyte-mediated immune responses. These changes in monocyte function may contribute to the persistent inflammation that characterizes prolonged labor^[41,42]^.

Monocyte dysregulation in prolonged labor is also marked by changes in the monocyte subtypes and their functional status. Typically, monocytes are classified into three subsets based on surface markers: classical (CD14++CD16−), intermediate (CD14++CD16 +), and nonclassical (CD14+CD16++). Each subset has distinct functional roles in inflammation and tissue remodeling. In prolonged labor, there is often an increased proportion of intermediate and nonclassical monocytes, which are more prone to producing pro-inflammatory cytokines and contributing to tissue damage. This shift in monocyte subsets may contribute to the excessive inflammatory response observed in prolonged labor. Additionally, a heightened activation of these monocytes, indicated by increased expression of HLA-DR and CD16, may lead to the sustained release of cytokines that interfere with normal uterine contractions and cervical ripening[4]. The role of monocyte-derived cytokines in regulating the inflammatory environment during labor highlights the importance of maintaining a balanced immune response. Monocyte dysfunction can exacerbate the inflammatory response, leading to tissue damage and impaired labor progression. Dysregulated cytokine production may not only disrupt uterine contractility but also compromise the cervix’s ability to soften and dilate. The increased persistence of these inflammatory mediators may lead to dysfunctional labor patterns, including uterine inertia, cervical arrest, and failure of labor to progress, which often result in the need for cesarean delivery[43]. Furthermore, the role of cytokine imbalances extends beyond monocytes and includes their interactions with other immune cells, such as neutrophils and T-cells. These interactions further perpetuate the inflammatory cycle, reinforcing monocyte-driven cytokine storms that hinder labor progression. For example, elevated levels of IL-6 and TNF-α can activate neutrophils and increase the production of reactive oxygen species, leading to cellular damage and further exacerbation of the inflammatory response. The combined effects of monocytes, neutrophils, and other immune mediators create a feedback loop that can sustain and amplify the inflammatory environment, contributing to labor complications such as prolonged labor (Table 1)[44].

**Table 1:** Monocyte subsets, cytokine profiles, and functional roles in normal labor versus prolonged labor

Monocyte subset	Surface markers	Cytokine profile	Functional role in normal labor	Observed alterations in prolonged labor
Classical	CD14++CD16−	TNF-α, IL-1β, IL-6, CCL2	Highly pro-inflammatory; initiates cervical ripening and myometrial activation; recruits neutrophils	Reduced proportion and delayed cytokine release; impaired initiation of labor cascade
Intermediate	CD14++CD16+	IL-6, IL-10, TNF-α	Antigen presentation; modulates balance between pro- and antiinflammatory responses; supports tissue remodeling	Dysregulated cytokine secretion; may contribute to delayed cervical remodeling
Nonclassical	CD14+CD16++	IL-10, TGF-β	Patrolling monocytes; endothelial surveillance; tissue repair; antiinflammatory regulation	Elevated proportion; delayed transition to pro-inflammatory phenotype; may impede effective labor progression

## Molecular signals in cervical ripening and myometrial activation

The initiation and progression of labor are orchestrated by a highly coordinated network of molecular signals, encompassing cytokines, chemokines, and MMPs, which together mediate cervical remodeling and myometrial contractility. These pathways are tightly regulated and largely driven by immune cell activation, particularly monocytes, which act as central modulators of the inflammatory cascade[45]. Pro-inflammatory cytokines, secreted primarily by classical monocytes and macrophages, play a pivotal role in labor. TNF-α and IL-1β induce the production of PGs in cervical stromal cells and the myometrium, directly stimulating uterine contractions. IL-6 amplifies local inflammation, promotes leukocyte recruitment, and enhances the expression of adhesion molecules in cervical tissue, facilitating the infiltration of additional immune cells. Antiinflammatory cytokines such as IL-10 and transforming growth factor-beta (TGF-β), secreted by nonclassical monocytes and regulatory immune cells, provide a counterbalance, limiting excessive tissue damage and ensuring controlled remodeling^[46,47]^.

Chemokines serve as critical chemotactic signals, guiding immune cells to the maternal-fetal interface. Monocyte chemoattractant protein-1 (CCL2) recruits monocytes to the cervix and myometrium, while CXCL8 (also known as IL-8) facilitates neutrophil migration into cervical tissues. These infiltrating leukocytes contribute to the inflammatory milieu and amplify local cytokine production, creating a positive feedback loop that accelerates cervical ripening and myometrial activation. The precise temporal and spatial expression of chemokines ensures that immune cell recruitment occurs in a coordinated manner, preventing premature or ineffective labor^[48,49]^. MMPs are enzymes responsible for ECM remodeling, a crucial process in cervical ripening. MMP-1, MMP-8, and MMP-9 degrade collagen and other ECM components, leading to cervical softening, effacement, and dilation. Monocyte-derived cytokines, particularly IL-1β and TNF-α, induce MMP expression in cervical fibroblasts and epithelial cells. Additionally, MMP activity is regulated by tissue inhibitors of metalloproteinases (TIMPs), ensuring that ECM degradation occurs in a controlled fashion to prevent excessive tissue injury^[50,51]^.

The interplay between cytokines, chemokines, and MMPs creates a self-amplifying inflammatory network that drives the key physiological events of labor. Monocytes orchestrate this process by secreting cytokines that stimulate PG production, recruiting additional immune cells via chemokines, and indirectly promoting ECM remodeling through MMP induction. In the myometrium, these signals enhance gap junction formation and calcium sensitivity, increasing uterine contractility and coordinating rhythmic contractions necessary for effective labor^[52,53]^. Disruptions in this molecular network, such as delayed monocyte activation, imbalanced cytokine secretion, or insufficient MMP activity, can impair cervical ripening and myometrial contraction, contributing to prolonged labor or labor dystocia. Understanding these molecular pathways provides a foundation for identifying predictive biomarkers and potential therapeutic targets to improve labor outcomes[54].

## Molecular signals in cervical ripening and myometrial activation

The initiation and progression of labor are orchestrated by a highly coordinated network of molecular signals, encompassing cytokines, chemokines, and MMPs, which together mediate cervical remodeling and myometrial contractility. These pathways are tightly regulated and largely driven by immune cell activation, particularly monocytes, which act as central modulators of the inflammatory cascade[45]. Pro-inflammatory cytokines, secreted primarily by classical monocytes and macrophages, play a pivotal role in labor. TNF-α and IL-1β induce the production of PGs in cervical stromal cells and the myometrium, directly stimulating uterine contractions. IL-6 amplifies local inflammation, promotes leukocyte recruitment, and enhances the expression of adhesion molecules in cervical tissue, facilitating the infiltration of additional immune cells. Antiinflammatory cytokines such as IL-10 and TGF-β, secreted by nonclassical monocytes and regulatory immune cells, provide a counterbalance, limiting excessive tissue damage and ensuring controlled remodeling^[46,47]^.

Chemokines serve as critical chemotactic signals, guiding immune cells to the maternal-fetal interface. Monocyte chemoattractant protein-1 (CCL2) recruits monocytes to the cervix and myometrium, while CXCL8 (also known as IL-8) facilitates neutrophil migration into cervical tissues. These infiltrating leukocytes contribute to the inflammatory milieu and amplify local cytokine production, creating a positive feedback loop that accelerates cervical ripening and myometrial activation. The precise temporal and spatial expression of chemokines ensures that immune cell recruitment occurs in a coordinated manner, preventing premature or ineffective labor^[48,49]^. MMPs are enzymes responsible for ECM remodeling, a crucial process in cervical ripening. MMP-1, MMP-8, and MMP-9 degrade collagen and other ECM components, leading to cervical softening, effacement, and dilation. Monocyte-derived cytokines, particularly IL-1β and TNF-α, induce MMP expression in cervical fibroblasts and epithelial cells. Additionally, MMP activity is regulated by TIMPs, ensuring that ECM degradation occurs in a controlled fashion to prevent excessive tissue injury^[50,51]^.

**Figure 2.**
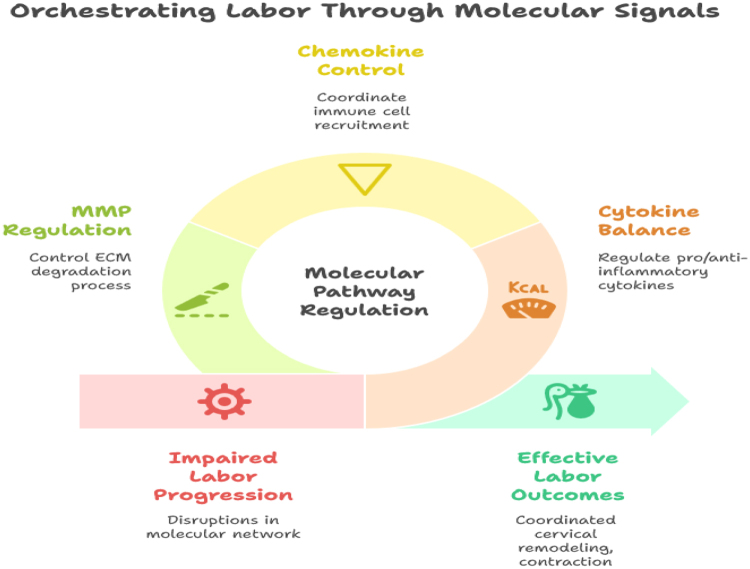
Molecular signals in cervical ripening and myometrial activation.

The interplay between cytokines, chemokines, and MMPs creates a self-amplifying inflammatory network that drives the key physiological events of labor. Monocytes orchestrate this process by secreting cytokines that stimulate PG production, recruiting additional immune cells via chemokines, and indirectly promoting ECM remodeling through MMP induction. In the myometrium, these signals enhance gap junction formation and calcium sensitivity, increasing uterine contractility and coordinating rhythmic contractions necessary for effective labor^[52,53]^. Disruptions in this molecular network, such as delayed monocyte activation, imbalanced cytokine secretion, or insufficient MMP activity, can impair cervical ripening and myometrial contraction, contributing to prolonged labor or labor dystocia. Understanding these molecular pathways provides a foundation for identifying predictive biomarkers and potential therapeutic targets to improve labor outcomes (Fig. 2)[53].

## Current and future directions for monocyte-based biomarkers in prolonged labor

Monocytes, as central players in the immune response during pregnancy and labor, have emerged as promising biomarkers for identifying and managing prolonged labor. Their ability to produce a wide range of cytokines and modulate inflammation makes them crucial in the processes of cervical ripening and uterine contractility, which are necessary for normal labor progression. The study of monocyte-based biomarkers in prolonged labor holds potential for improving our understanding of the underlying mechanisms of labor dysfunction and could lead to novel diagnostic and therapeutic strategies^[54,55]^.

## Current insights and methodologies

Currently, research on monocyte-based biomarkers in prolonged labor largely focuses on the profiling of monocyte subsets and their cytokine production profiles. Flow cytometry is a widely used technique to assess the expression of surface markers (e.g., CD14, CD16, and HLA-DR) on monocytes, which provides insight into their activation status and potential contribution to labor. Additionally, cytokine assays (such as ELISA, PCR, and multiplex assays) are employed to measure the levels of key pro-inflammatory cytokines (e.g., IL-1β, TNF-α, and IL-6) in maternal blood and cervicovaginal secretions. These methodologies have shown that women with prolonged labor often exhibit altered monocyte subset distributions and higher levels of pro-inflammatory cytokines compared to those with normal labor progression[56]. Despite the progress, several challenges remain in establishing monocytes as reliable biomarkers for prolonged labor. The heterogeneity of monocytes, with their diverse subsets and varying activation states, complicates their role as universal markers. Furthermore, the dynamics of cytokine production and the interplay between various immune cells in labor complicate the interpretation of findings. Research has yet to establish standardized protocols for sample collection, processing, and analysis, which limits the reproducibility and comparability of results across studies. Nevertheless, current research highlights that alterations in monocyte function, including dysregulated activation and cytokine production, may contribute significantly to prolonged labor[57].

## Conclusion

Monocytes play a critical role in the immune response during pregnancy and labor, and their dysregulation can contribute significantly to complications such as prolonged labor. The dynamic involvement of monocytes in cervical ripening, uterine contractility, and inflammatory processes highlights their potential as biomarkers for predicting and managing labor progression. Despite advances in understanding monocyte-driven mechanisms, significant challenges remain in establishing reliable biomarkers for clinical use. The heterogeneity of monocyte subsets, the complexity of cytokine interactions, and the need for standardized methodologies all present hurdles that must be overcome to fully realize the potential of monocyte-based biomarkers. Current research has identified key cytokines and markers associated with monocyte activation that could provide valuable insight into labor progression. Emerging technologies such as single-cell RNA sequencing and miRNA profiling offer exciting opportunities to explore the molecular underpinnings of monocyte function during labor and identify more specific and precise biomarkers. These advances, combined with novel therapeutic approaches targeting monocyte regulation, have the potential to revolutionize the management of prolonged labor, leading to improved maternal and neonatal outcomes.

## Data Availability

Not applicable as this is a Narrative Review.

## Research registration unique identifying number (UIN)

Not applicable as this a Narrative Review.
